# Supplementary material for: Electrostatic catalysis of a click reaction in a microfluidic cell
Source: Nat Commun. 2024 Jan 26;15:790. doi: 10.1038/s41467-024-44716-2 (PMC10817948; doi:10.1038/s41467-024-44716-2)
Supplement: Supplementary file 1 — Supplementary Information [file 41467_2024_44716_MOESM1_ESM.pdf]

Supplementary Information

Electrostatic catalysis of a click reaction in a microfluidic cell

Semih Sevim[1], Roger Sanchis-Gual[1], Carlos Franco[1], Albert C. Aragonès[2], Nadim Darwish[3], Donghoon Kim[1], Rosaria Anna Picca[4], Bradley J. Nelson[1], Eliseo Ruiz[5], Salvador Pané[1]*, Ismael Díez-Pérez[6]*, Josep Puigmartí-Luis[2,7]*

[1]*Institute of Robotics and Intelligent Systems, ETH Zurich, Tannenstrasse 3, CH-8092 Zurich, Switzerland.*
[2]*Departament de Ciència de Materials i Química Física, Institut de Química Teòrica i Computacional, University of Barcelona (UB), Marti i Franquès 1, 08028 Barcelona, Spain .*
[3]*School of Molecular and Life Sciences, Curtin University, Bentley 6102, Western Australia, Australia.*
[4]*Chemistry Department, University of Bari "Aldo Moro", via E. Orabona 4, 70125 Bari, Italy.*
[5]*Departament de Química Inorgànica i Orgànica, Institut de Química Teòrica i Computacional, University of Barcelona (UB), Diagonal 645, 08028 Barcelona, Spain.*
[6]*Department of Chemistry, Faculty of Natural, Mathematical & Engineering Sciences, King's College London, Britannia House, 7 Trinity Street, London SE1 1DB (United Kingdom).*
[7]*Institució Catalana de Recerca i Estudis Avançats (ICREA), Pg. Lluís Companys 23, 08010 Barcelona, Spain.*

*e-mail: *vidalp@ethz.ch, ismael.diez_perez@kcl.ac.uk, josep.puigmarti@ub.edu*

Table of Contents

Supplementary Figures 3

Supplementary Tables 12

Supplementary Notes 13

Supplementary Note 1: Materials 13

Supplementary Note 2: Preparation of gold-coated glass slides 13

Supplementary Note 3: Functionalization of gold-coated slides 13

Supplementary Note 4: Electrochemical characterizations 14

Supplementary Note 5: X-ray photoelectron spectroscopy (XPS) 16

Supplementary Note 6: Microfluidic experimental procedure using toluene as a solvent (low polar medium case) 16

Supplementary References 18

Supplementary Figures

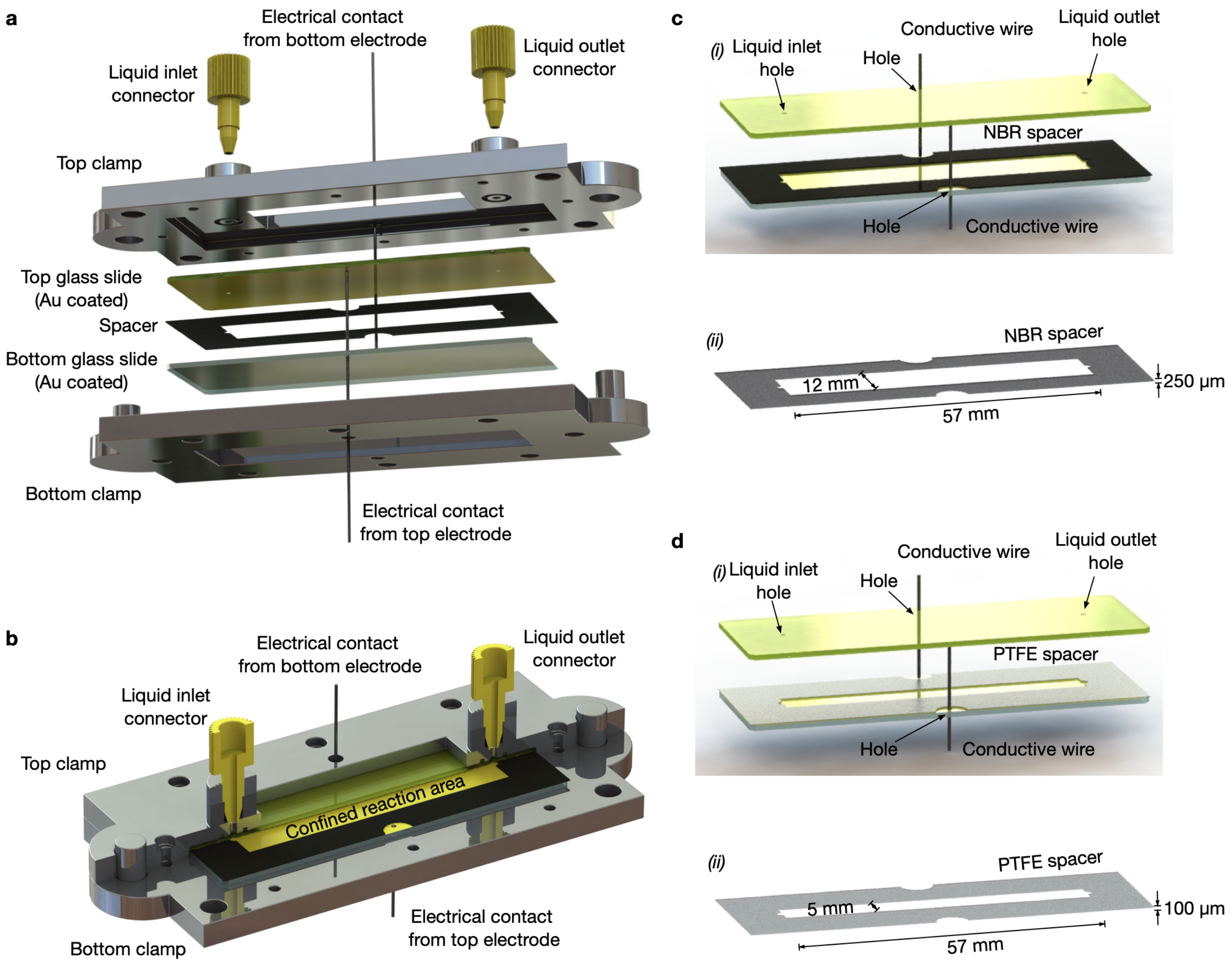

Fig. S1. Design of microfluidic cell. a-b Schematic drawing showing split version of the microfluidic cell prior to its assembly to represent each part separately (in panel a) and assembled version of the microfluidic cell with a section-cut in the top part to demonstrate the confined reaction area formed with a spacer between two gold-coated electrodes (in panel b). **c-d** Isolated schematic view of (i) gold-coated glass slides showing the hole locations and (ii) spacer showing the confined reaction area dimensions used with acetonitrile and toluene, respectively in c and d. Note that top slide has three holes (i.e., two for the liquid inlet and outlet, and one for passing a conductive wire to make the electrical contact to the bottom slide), whereas the bottom slide has only one hole (for passing a conductive wire and make the electrical contact to the top slide).

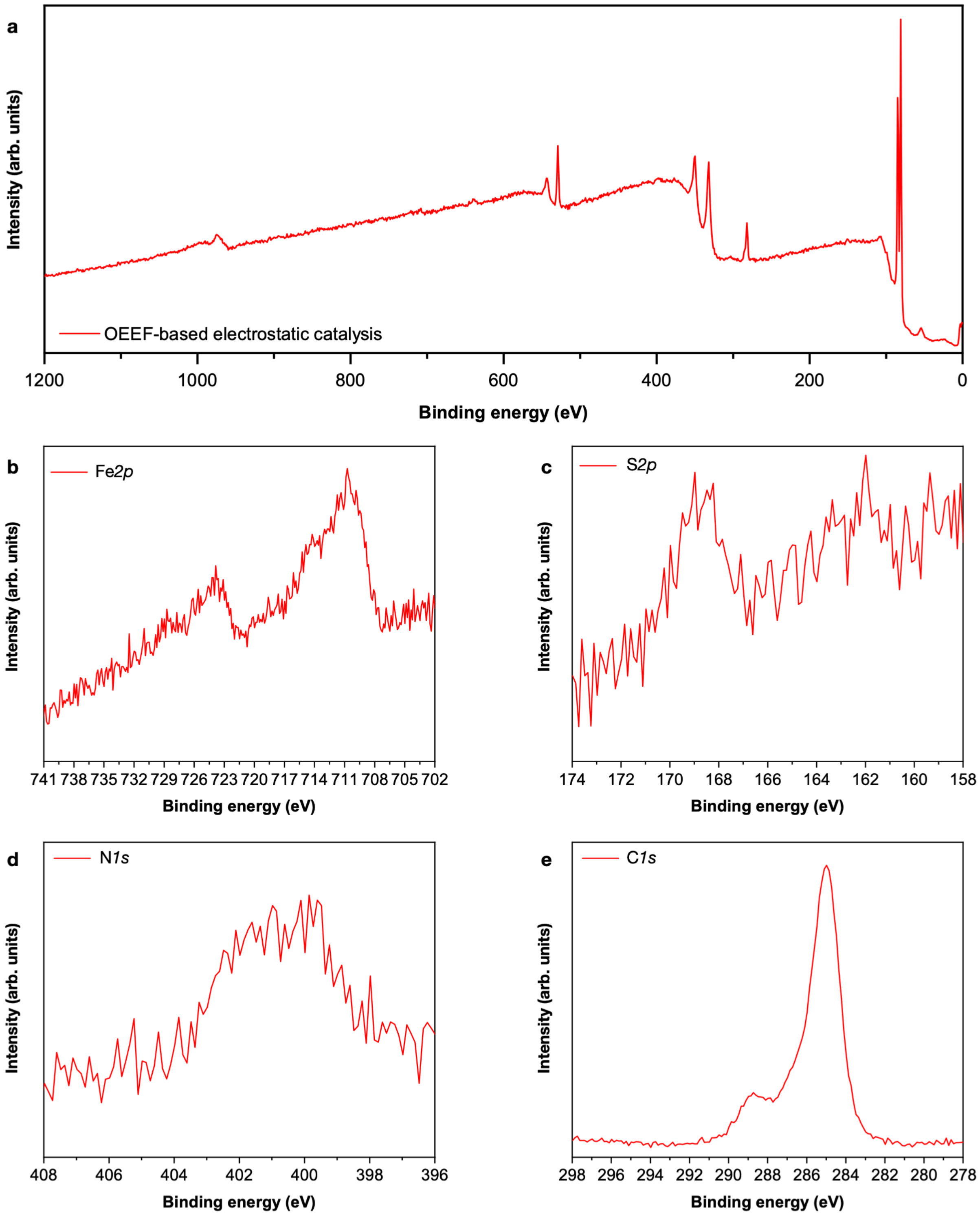

Fig. S2. Chemical state analysis of the sample prepared with electrostatic catalysis. a Complete XPS spectrum of the sample prepared with electrostatic catalysis. **b-e** Corresponding high-resolution XPS spectrum showing the Fe*2p*, S*2p*, N*1s*, C*1s* regions respectively from b to e.

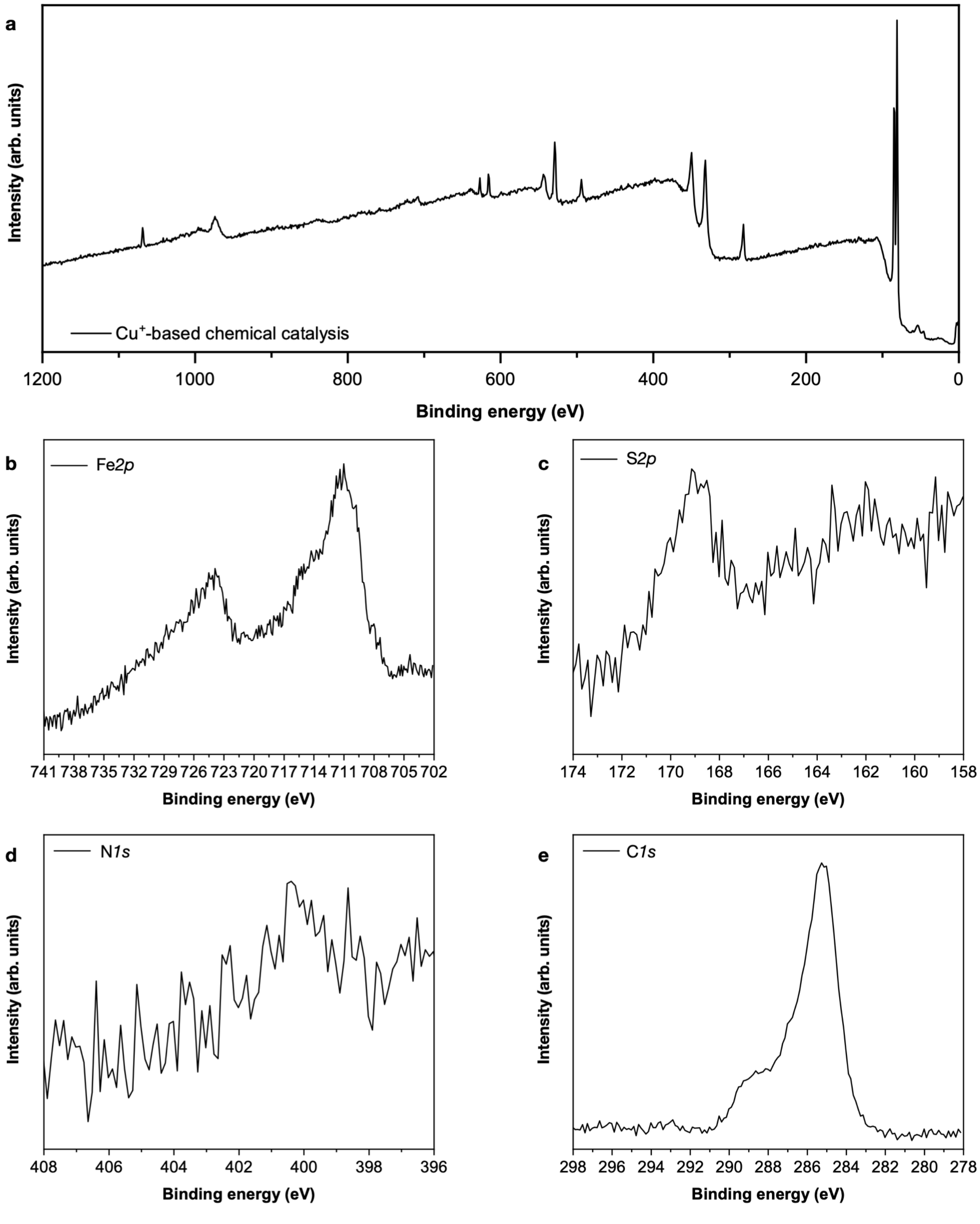

Fig. S3. Chemical state analysis of the sample prepared with Cu^{+}-based chemical catalysis. a Complete XPS spectrum of the sample prepared with Cu^{+}-based chemical catalysis. **b-e** Corresponding high-resolution XPS spectrum showing the Fe*2p*, S*2p*, N*1s*, C*1s* regions respectively from b to e.

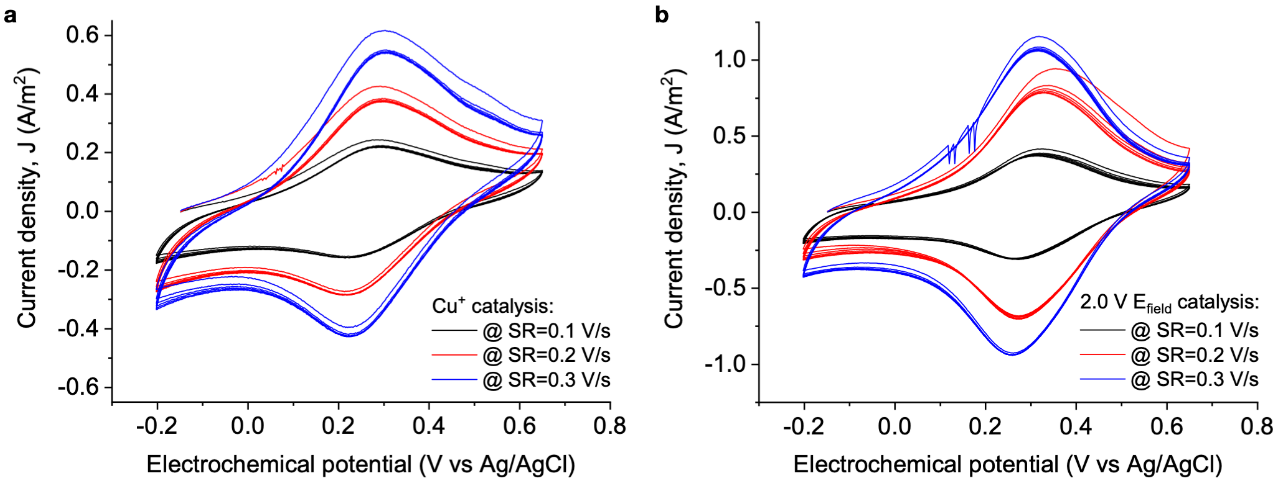

Fig. S4. CV results with respect to different scan rates. a-b Multiple CV cycles at different scan rates (SR), such as 0.1 V s^{-1} (black), 0.2 V s^{-1} (red), 0.3 V s^{-1} (blue) performed on the sample prepared with Cu^{+}-based chemical catalysis (in panel a) and electrostatic catalysis with a bias voltage of 2 V (in panel b). Stability in the oxidation-reduction peaks within multiple cycles indicates that ferrocene is chemically attached to the functionalized gold electrode via the azide-alkyne cycloaddition.

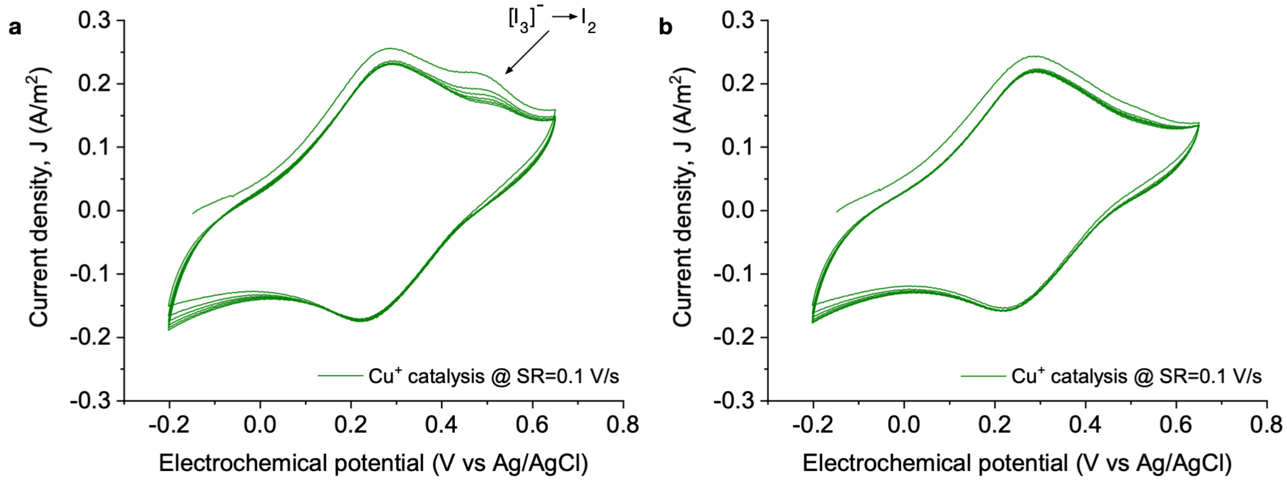

Fig. S5. Surface contaminations during Cu^+-based chemical catalysis. a CV of the sample prepared with Cu^+-based chemical catalysis at a scan rate (SR) of 0.1 V s^{-1} showing an additional peak corresponding the oxidation of the precipitated iodide ($[I_3]^- \rightarrow I_2$)[1]. Note that this oxidation peak is diminishing with consecutive cycles due to detachment of physiosorbed molecules from the electrode substrate, whereas the peaks that correspond to the oxidation and reduction of the chemically attached ferrocene are stable. **b** CV of the same sample presented in a, after the physiosorbed materials are completely removed.

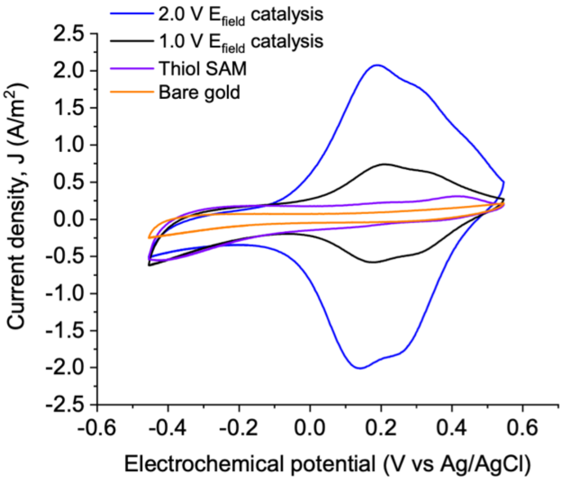

Fig. S6. Control experiments for electrostatic catalysis and effect of surface functionalization. CV of the bare-gold electrode (orange) and the functionalized (azide-terminated) electrode (purple). Oxidation-reduction of ferrocene attached to azide-terminated electrode via azide-alkyne cycloaddition catalyzed by OEEF at a bias voltage of 1 V (black) and 2 V (blue). Note that, these repetition experiments were performed with functionalized electrodes prepared in a new batch. Due to the likely differences in the formation of functional groups on the surface (i.e. disordered arrangement) and supramolecular interaction between cyclopentadienyl groups[2,3], the shape and area of the voltammetric peaks are slightly different than the ones presented in Fig. 2a. However, the effect of applied bias voltage is consistent with the results presented in the manuscript, i.e., 2 V applied bias voltage results in a much higher yield of electrostatically catalyzed click reaction than the sample prepared with 1 V bias voltage by employing functionalized electrodes from the same batch. The cyclic voltammograms are obtained at a scan rate of 0.3 V s^{-1}.

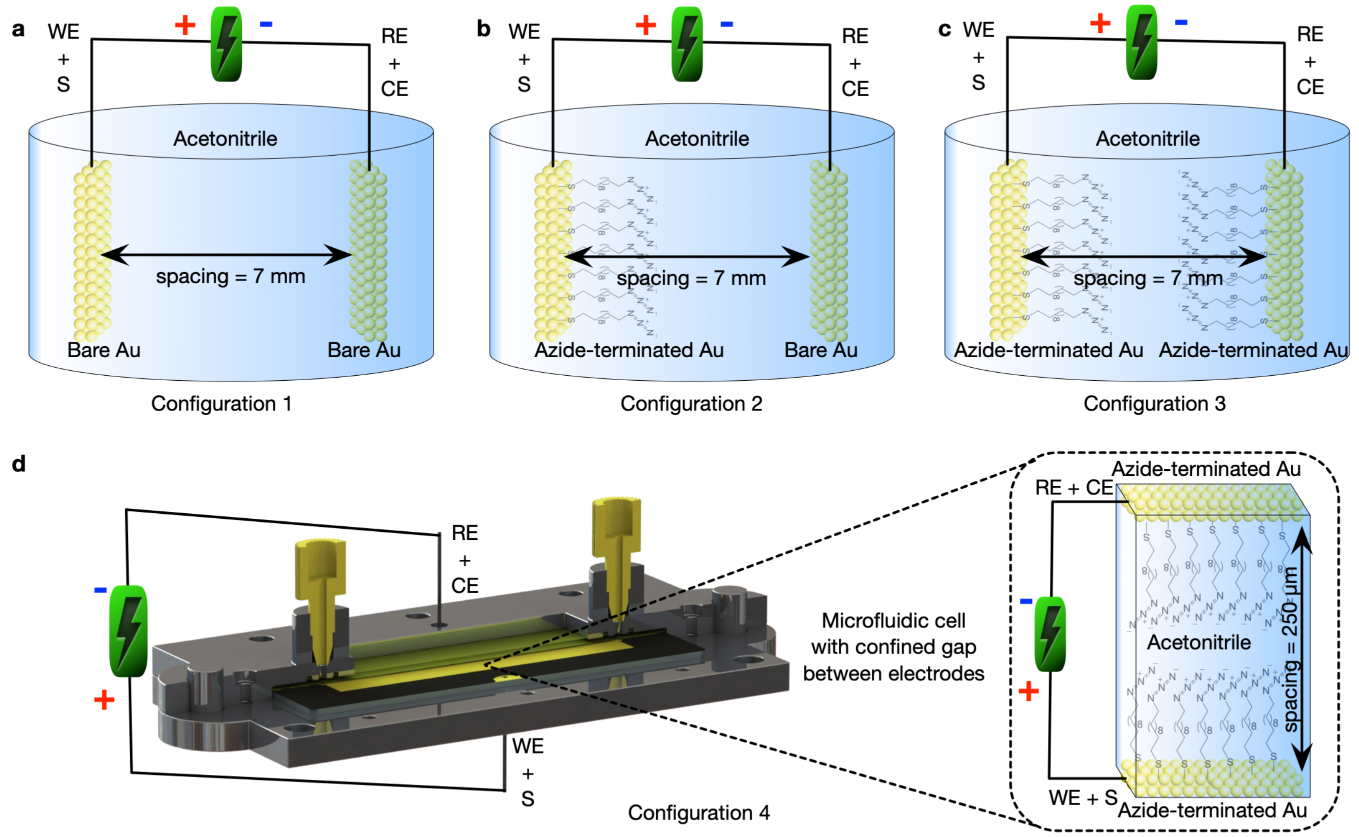

Fig. S7. Electrochemical cell configurations for Chronoamperometry (CA) measurements. a-c Different electrode configurations in a beaker containing pure acetonitrile, such as **a** two parallel bare gold electrodes; **b** an azide-terminated gold electrode against a bare gold electrode; **c** two parallel azide-terminated electrodes. **d** Two parallel azide-terminated electrodes confined in microfluidic cell. All CA measurements were performed with two-electrode configuration (without using any reference electrode) and in pure acetonitrile (as an electrolyte medium). In the experiments where the glass beaker was used as an electrochemical cell, the distance between the working and counter electrodes was set to be 7 mm, whereas in the microfluidic electrochemical cell configuration the distance was set to be 250 µm.

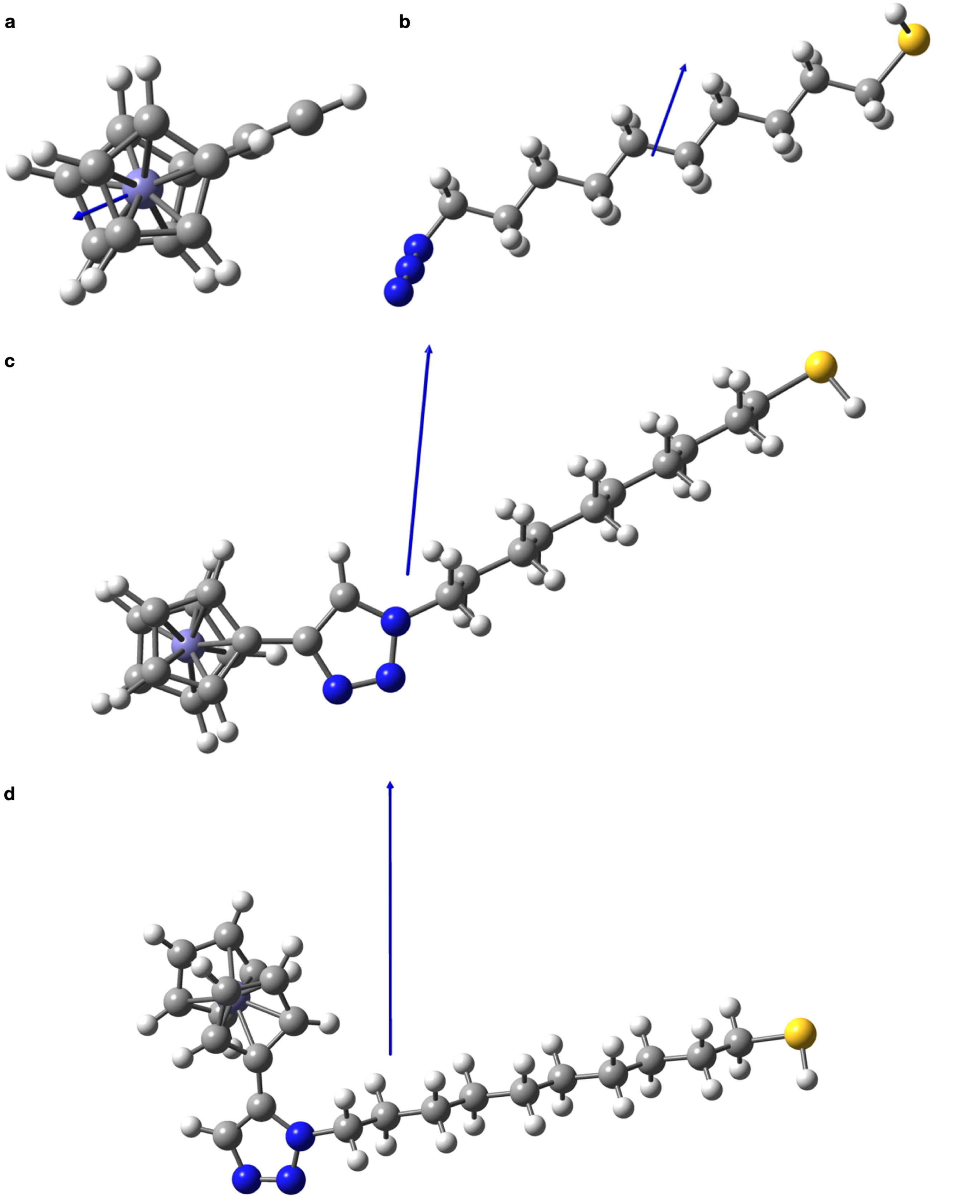

Fig. S8. Calculated electrical dipole moments at DFT level for the three molecular systems. **a-b** Show the magnitude and orientation for reactants (fcac and c10n3, respectively). **c-d** show the two plausible products (1,4-isomer and 1,5-isomer for fcacn3c10, respectively).

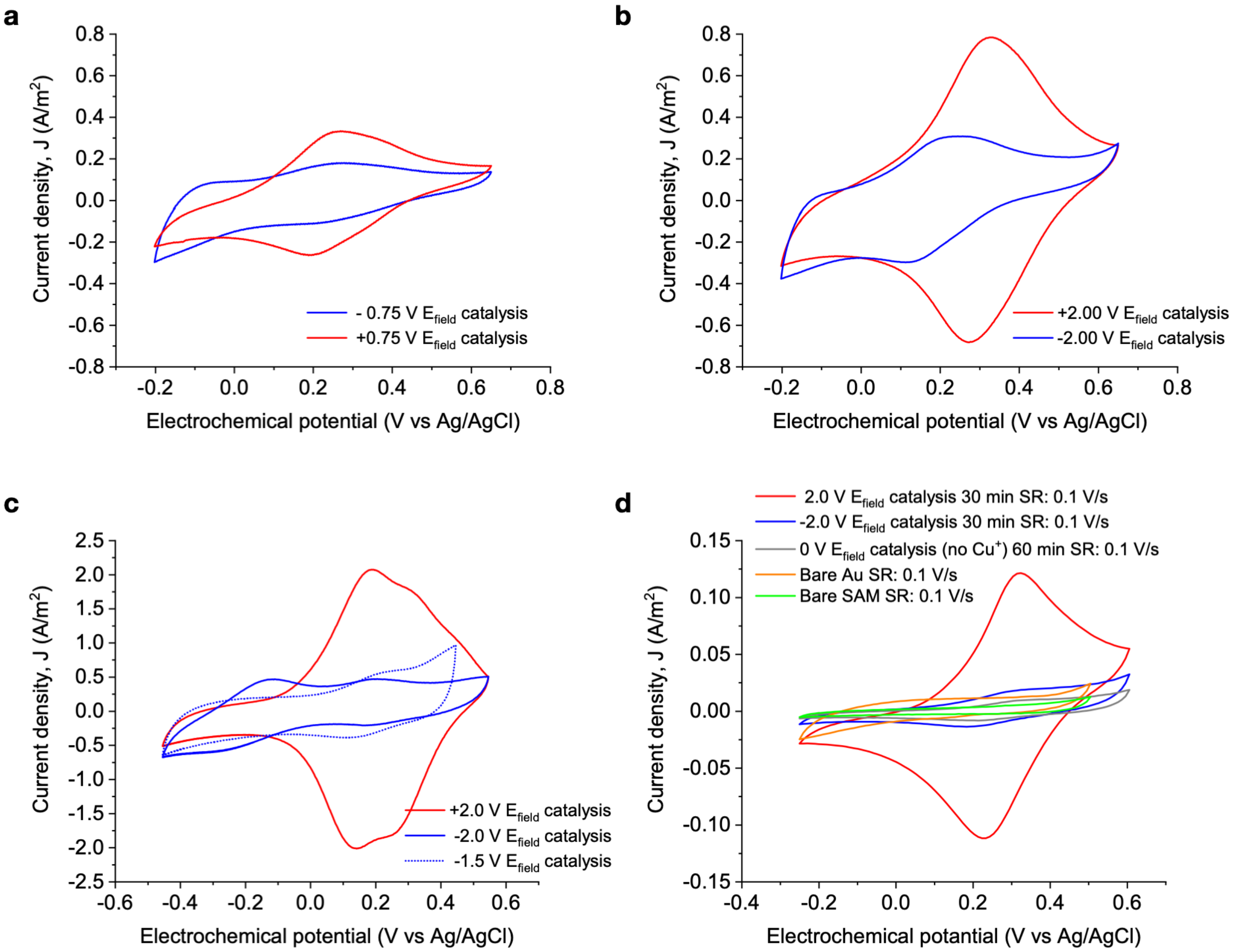

Fig. S9. Ferrocene (Fc) voltametric signals of electrostatic catalysis performed at different polarities. Comparing the results obtained at **a** ±0.75 V bias voltages (red and blue, respectively); and **b** ±2.00 V bias voltages (red and blue, respectively) from the same sample batch. **c** shows results obtained from a different batch performed at ±2.00 V (red and blue solid line, respectively) and -1.50 V (blue dotted line) bias voltages. **d** shows repetition experiments from different surface functionalization batches at ±2.00 V (red and blue, respectively) together with control groups, i.e. in the absence of both electric field (0 V) and Cu^+ catalysis (grey), bare-gold electrode (orange), and the functionalized (azide-terminated) electrode (green). The cyclic voltammograms in a and b were obtained at a scan rate (SR) of 0.2 V s^{-1}, in c and d obtained at a scan rate (SR) of 0.3 V s^{-1} and 0.1 V s^{-1}, respectively.

Supplementary Tables

Table S1. Surface chemical composition of the samples prepared via click reaction. The atomic percentage of the elements detected by XPS of the samples prepared by either Cu^{+}-based chemical catalysis or electrostatic catalysis is reported, as mean value ± 1s (n = 3).

Sample	C%	N%	O%	Na%	S%	K%	Fe%	I%	Au%
Cu^{+} catalysis	42.9±1.2	0.9±0.5	26.1±1.2	5.8±0.5	1.1±0.5	≤ 0.2	4.0±0.4	1.1±0.5	17.9±0.2
Electrostatic catalysis	51±5	3.2±0.4	24±3	-	1.0±0.5	-	2.4±0.4	-	18.4±1.5

Table S2. Theoretical dipole moment calculations. Calculated dipole values (in Debyes) for the molecular system in vacuum and using a continuum model to simulate the solvents (toluene and acetonitrile, see Computational details in Methods). The $\Delta\mu$ magnitude corresponds to the variation in the dipolar moment between reactants (fcac and c10n3) and the two plausible products (1,4-isomer and 1,5-isomer for fcacn3c10, respectively) of the click reaction.

	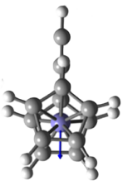	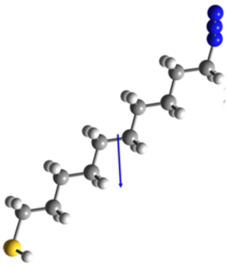	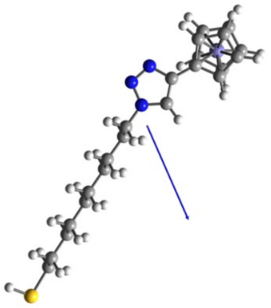		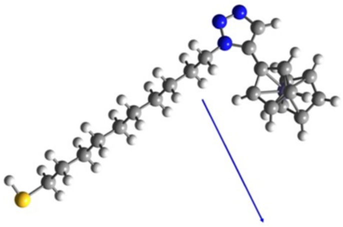	
	fcac	c10n3	fcacn3c10 1,4-isomer	Δμ	fcacn3c10 1,5-isomer	Δμ
vacuum	1.0115	1.2954	2.8305	+0.5235	4.1467	+1.839
toluene	1.2641	1.3777	3.5268	+0.8850	4.8968	+2.255
acetonitrile	1.5387	1.4306	4.3079	+1.3386	5.6164	+2.647

Supplementary Notes

Supplementary Note 1: Materials

Bis(11-azidoundecyl) disulfide 99% (HPLC), Ethynylferrocene 97%, Copper(I) iodide-triethyl phosphite, Tetrabutylammonium perchlorate ≥99.0% (for electrochemical analysis), Acetonitrile (HPLC grade) ≥99.9%, Toluene ACS reagent ≥99.5%, Ethanol (absolute) ≥99.8%, Acetone ≥99.8% were purchased Sigma-Aldrich (Sigma-Aldrich Chemie GmbH, Switzerland). Hydrogen peroxide solution >30% (w/w) in H_2O and Sulfuric acid >95% were bought from Acros Organics (Fisher Scientific AG, Switzerland). Isopropyl alcohol >99.7% was purchased from VWR International AG (Switzerland). All reagents were used without additional purification.

Supplementary Note 2: Preparation of gold-coated glass slides

First, commercial microscope slides (75.5 mm x 25.5 mm, Thermo Scientific™ Microscope Slides, Ground 45°, Fisher Scientific AG, Switzerland) were drilled using a micro milling machine (MF 70, Proxxon GmbH, Germany) with a diamond tip to create holes (1 mm in diameter) for the inlet, outlet and electrical contacts. Specifically, the top glass slide has three holes, i.e., two holes for the liquid inlet and outlet to achieve continuous-flow of the reagent in the microfluidic cell, and one hole as an opening to take the electrical contact from the bottom electrode. On the other hand, the bottom glass slide has only one hole which is used as an opening to take the electrical contact from the top electrode (see Design of the microfluidic cell in Methods). Drilled glass slides were cleaned with acetone, isopropanol, and Milli-Q water, and dried with Nitrogen (N_2) gas prior to the metallization step. Then, they were coated with chromium (10 nm) as an adhesion layer and subsequently with gold (100 nm) as the electrode layer using an electron-beam evaporation system.

Supplementary Note 3: Functionalization of gold-coated slides

Prior to the functionalization of the gold-coated glass slides with azide-terminated thiol, all the glassware was cleaned using piranha solution (3:1 mixture of sulfuric acid, H_2SO_4 and hydrogen peroxide, H_2O_2) and vigorously rinsed with freshly prepared Milli-Q water to remove residual piranha solution. After the washing step with Milli-Q water, the gold-coated slides were sonicated in ethanol and dried with N_2 gas. The functionalization was performed by immersing the gold-coated glass slides in 1 mM ethanolic solution of Bis(11-azidoundecyl) disulfide for 24 hours at room temperature and under a N_2-saturated environment. Note that, to avoid the oxidation of the thiol, the ethanol was bubbled with N_2 gas (for ≥20 minutes) before the preparation of the solution used for the functionalization of the gold-coated glass slides. After the functionalization, the derivatized slides were immediately rinsed with ethanol to remove the residual of Bis(11-azidoundecyl) disulfide solution.

Supplementary Note 4: Electrochemical characterization

All electrochemical measurements, e.g., cyclic voltammetry (CV), square-wave voltammetry (SWV) and chronoamperometry (CA), were performed with Autolab/PGSTAT204 (Metrohm AG, Switzerland) using NOVA 2.0 software.

Cyclic voltammetry (CV) and square-wave voltammetry (SWV). After the preparation of the samples (with either electrostatic or Cu^+-based chemical catalysis) in the microfluidic cell, the working electrodes (azide-terminated gold coated slides) were characterized in a separate electrochemical cell to a posteriori interrogate the yield of the click reaction by investigating the ferrocene attachment via azide-alkyne cycloaddition. The electrochemical cell was built in a glass beaker (50 mL) including the sample slide from the microfluidic experiment, a platinum (Pt)-coated glass slide and a silver wire as the working, counter and reference electrodes, respectively. The electrolyte solution (0.1 M tetrabutylammonium perchlorate in acetonitrile) was filled in electrochemical cell until the half of the area of the working electrode was immersed in the electrolyte solution, and then the electrochemical cell was bubbled with N_2 for 5 minutes. All the electrochemical measurements were performed with an Autolab/PGSTAT204 (Metrohm AG, Switzerland) using the NOVA 2.0 software. Prior to the electrochemical characterization of each sample, the electrolyte solution was changed with a fresh one, the reference and counter electrodes were cleaned with acetone and dried with N_2. Note that, the cyclic and square-wave voltammograms were recorded using a silver (Ag) wire as a reference electrode and then the electrochemical potential was corrected with respect to the Ag/AgCl standard reference electrode by measuring the open circuit potential of the Ag wire with respect to the Ag/AgCl standard reference electrode.

Chronoamperometry (CA) for characterization of localized electric field near electrode surfaces. In order to characterize the electric field near the electrode surface CA measurements were performed. To mimic the experimental conditions for the electrostatic catalysis, a two-electrode configuration was used in CA measurements. More specifically, the electrochemical cell was configurated with only working and counter electrodes (i.e., without any reference electrode), and the pure acetonitrile solution is used as the electrolyte medium. Three different electrochemical cell arrangements were tested in a glass beaker (50 mL) (Supplementary Figs. 7a-c), i.e., (1st configuration) bare gold surface vs bare gold surface (Supplementary Fig. 7a), (2nd configuration) azide-terminated gold surface vs bare gold surface (Supplementary Fig. 7b) and (3rd configuration) azide-terminated gold surface vs azide-terminated gold surface (Supplementary Fig. 7c) as working vs counter electrodes, respectively. Moreover, the 4th electrochemical cell configuration was used to investigate the OEEF localized near electrode surfaces during the electrostatic catalysis experiments in our microfluidic cell (Supplementary Fig. 7d). To this end, the microfluidic cell was assembled by clamping a 250 µm-thick NBR spacer between two azide-terminated electrodes and filled with pure acetonitrile. After the microfluidic cell was completely filled with pure acetonitrile, the flow was stopped. All CA measurements were performed by starting from measured open circuit potential (V_{OCP}) and followed by applying a step voltage ($V_{applied}$, ranging

between 0.25 V and 2 V) to the parallel electrodes for charging the formed electrochemical capacitor and subsequently discharging it at the open circuit potential (V_{OCP}). Note that V_{OCP} were chosen to be the initial potential for CA measurements for high reproducibility and less hysteresis[4,5]. The charging/discharging response is associated with total capacitance on the electrode surface which is due to the double layer formation of pure acetonitrile and the azide-terminated functionalization layer on the gold surface (in case it is functionalized).

These above-mentioned configurations and experimental methodology allowed us to measure the current transient during the charging and discharging of the electrochemical circuit which can be modelled as a RC circuit to obtain the time constant[4]. More specifically, as charging progress current decreases exponentially (Eq. (1)), while charge is increasing (Eq. (2)) with following equations:

$$I(t) = \frac{V_{applied}}{R} e^{-t/RC} \tag{1}$$

$$Q(t) = CV_{applied}(1 - e^{-t/RC}) \tag{2}$$

where $I(t)$ and $Q(t)$ are respectively the current and charge at time t, $V_{applied}$ is the bias voltage applied to the parallel electrodes, R is the resistance, C is the total capacitance associated with charge accumulated on the electrode surfaces. During charging the initial current, $I(0)$ at $t=0$ is the maximum current, I_{max} and could be obtained directly from the exponential fittings to CA measurements. Thus, the resistance of modelled circuit can be calculated with Eq. (3).

$$as\ t = 0, \qquad I(0) = I_{max} = \frac{V_{applied}}{R} \Longrightarrow R = \frac{V_{applied}}{I_{max}} \tag{3}$$

The exponential fitting to the CA data during charging progress also gives us the time constant, τ of the modelled circuit which is used to calculate total capacitance associated with charge as in Eq. (4).

$$\tau = RC \Longrightarrow C = \tau/R \tag{4}$$

As time goes to infinity, the accumulated charge in the capacitor reaches its maximum value, Q_{max} and could be obtained from Eq. (5). Finally, knowing the total charge associated with the electrochemical capacitor on the electrode surface allows us for the calculation of the localized electric fields near the electrode surface, E_{max} with the Gauss's law (Eq. (6)), where the ε_0 is the vacuum permittivity (8.85 x 10^{-12} F m^{-1}) and ε_r is the relative permittivity of medium (37.5).

$$as\ t \to \infty, \qquad \lim_{t\to\infty} Q(t) = Q_{max} = CV_{applied} \tag{5}$$

$$as\ t \to \infty, \qquad \lim_{t\to\infty} E(t) = E_{max} = \frac{Q_{max}}{\varepsilon_0 \varepsilon_r A} \tag{6}$$

Supplementary Note 5: X-ray photoelectron spectroscopy (XPS)

X-ray photoelectron spectroscopic characterization was performed with a PHI Versaprobe II spectrometer (Chanhassen, MN, USA) equipped with monochromatic Al Kα source (1486.6 eV, 200 µm spot size). Charge neutralization was constantly applied. First, survey scans were acquired on at least three points for each sample at a 117.4 eV pass energy (1.0 eV step) to identify all the elements present on the surface. Then, high-resolution spectra were registered at 58.7 eV pass energy (0.125 eV step) for quantification. Binding energy (BE) scale was corrected on Au$4f_{7/2}$ component at 84.0 eV. Multipak software (v. 9.9.0.8, ULVAC-PHI, Inc., Japan) was used for element quantification. Chemical composition is expressed in terms of surface atomic percentage (El%).

Supplementary Note 6: Microfluidic experimental procedure using toluene as a solvent (low polar medium case)

Note that all reagent solutions were filtered prior to injection of microfluidic device to avoid any precipitation in solution. Moreover, using toluene as a solvent requires to change the NBR spacer with a PTFE spacer due to solvent compatibility issues. Since the PTFE spacer (100 µm thick) is thinner and stiffer than NBR spacer (250 µm-thick), the width of reaction chamber was decreased from 12 mm to 5 mm in order to increase the sealing performance of spacer by increasing its surface area in contact with gold-coated glass slides.

Electrostatic catalysis under continuous-flow conditions. (i) The microfluidic cell was assembled by clamping a 100 µm-thick PTFE spacer between gold-coated glass slides (i.e., the azide-terminated working and counter electrode) to define a confined reaction area (57 mm x 5 mm) between the parallel electrodes (separation distance ca. 100 µm) (Supplementary Fig. 1d). (ii) The electrical contact for the top (or bottom) electrode was taken with a conductive wire going through the concentric holes present on the bottom (or top) clamp, bottom (or top) glass slide and spacer until touching to the top (or bottom) electrode surface (Supplementary Figs. 1a and d). (iii) Prior to sending the reagent solution (i.e., 0.1 M ethynylferrocene in toluene), the microfluidic cell was flushed with solvent (i.e., pure toluene) with a flow rate of 200 µL min^{-1} for 5 minutes. (iv) Then, 0.1 M ethynylferrocene in toluene was injected in the microfluidic cell (with a flow rate of 200 µL min^{-1} for ca. 2 minutes) to remove the solvent and quickly fill the reaction chamber with the reagent solution. (v) Once the reaction chamber was completely filled with the reagent solution, the flow rate was decreased to 50 µL min^{-1} and a constant DC bias voltage (1.5 V) was applied to the parallel electrodes

for a period of 30 minutes. (vi) After 30 minutes reaction time under the continuous-flow conditions, the applied voltage was stopped, and a pure toluene solution was injected (200 µL min^{-1} for 5 minutes) in the microfluidic cell to remove the excess ethynylferrocene and clean the electrode surfaces to prevent the presence of any physiosorbed molecules or surplus reactants. (vii) Finally, the microfluidic cell was dissembled, and the working electrodes were characterized in a separate electrochemical cell to a posteriori interrogate the yield of the click reaction (vide infra).

Cu^{+}-based chemical catalysis under continuous-flow conditions. The same experimental procedure, described for Electrostatic catalysis under continuous-flow condition, was repeated except for step (v). In this case, the reactant solution that was injected in the microfluidic cell contained Copper(I) iodide-triethyl phosphite (>20 mol% relative to the ethynylferrocene) and ethynylferrocene (0.1M) in toluene. The reaction was performed without applying a bias voltage (step (v)).

Supplementary References

1. Anari, E. H. B. *et al.* Substituted ferrocenes and iodine as synergistic thermoelectrochemical heat harvesting redox couples in ionic liquids. *Chem. Commun.* **52**, 745–748 (2016).
2. Duffin, T. J., Nerngchamnong, N., Thompson, D. & Nijhuis, C. A. Direct measurement of the local field within alkyl-ferrocenyl-alkanethiolate monolayers: Importance of the supramolecular and electronic structure on the voltammetric response and potential profile. *Electrochimica Acta* **311**, 92–102 (2019).
3. Zheng, Q. & Shao, H. Influence of intermolecular H-bonding on the acid-base interfacial properties of -COOH and ferrocene terminated SAM. *Journal of Electroanalytical Chemistry* **829**, 88–94 (2018).
4. Jitvisate, M. & Seddon, J. R. T. Direct Measurement of the Differential Capacitance of Solvent-Free and Dilute Ionic Liquids. *J. Phys. Chem. Lett.* **9**, 126–131 (2018).
5. Lockett, V., Sedev, R., Ralston, J., Horne, M. & Rodopoulos, T. Differential Capacitance of the Electrical Double Layer in Imidazolium-Based Ionic Liquids: Influence of Potential, Cation Size, and Temperature. *J. Phys. Chem. C* **112**, 7486–7495 (2008).
